# Development and validation of a nomogram model for predicting unfavorable functional outcomes in ischemic stroke patients after acute phase

**DOI:** 10.3389/fnagi.2023.1161016

**Published:** 2023-07-14

**Authors:** Chengjie Yan, Yu Zheng, Xintong Zhang, Chen Gong, Shibin Wen, Yonggang Zhu, Yujuan Jiang, Xipeng Li, Gaoyong Fu, Huaping Pan, Meiling Teng, Lingfeng Xia, Jian Li, Kun Qian, Xiao Lu

**Affiliations:** ^1^Department of Rehabilitation Medicine, The First Affiliated Hospital of Nanjing Medical University, Nanjing, China; ^2^Department of Neurology, Jiuquan City People’s Hospital, Jiuquan, China; ^3^Department of Rehabilitation Medicine, The First People’s Hospital of Lianyungang, Lianyungang, China; ^4^Department of Rehabilitation Medicine, Cangzhou Central Hospital, Cangzhou, China; ^5^Department of Neurology, Xingtai People’s Hospital, Xingtai, China; ^6^Department of Rehabilitation Medicine, The First People’s Hospital of Yibin, Yibin, China; ^7^Department of Rehabilitation Medicine, The Affiliated Jiangning Hospital of Nanjing Medical University, Nanjing, China

**Keywords:** nomogram, ischemic stroke, modified Rankin Scale, rehabilitation, predictive factor

## Abstract

**Introduction:**

Prediction of post-stroke functional outcome is important for personalized rehabilitation treatment, we aimed to develop an effective nomogram for predicting long-term unfavorable functional outcomes in ischemic stroke patients after acute phase.

**Methods:**

We retrospectively analyzed clinical data, rehabilitation data, and longitudinal follow-up data from ischemic stroke patients who underwent early rehabilitation at multiple centers in China. An unfavorable functional outcome was defined as a modified Rankin Scale (mRS) score of 3–6 at 90 days after onset. Patients were randomly allocated to either a training or test cohort in a ratio of 4:1. Univariate and multivariate logistic regression analyses were used to identify the predictors for the development of a predictive nomogram. The area under the receiver operating characteristic curve (AUC) was used to evaluate predictive ability in both the training and test cohorts.

**Results:**

A total of 856 patients (training cohort: *n* = 684; test cohort: *n* = 172) were included in this study. Among them, 518 patients experienced unfavorable outcomes 90 days after ischemic stroke. Trial of ORG 10172 in Acute Stroke Treatment classification (*p* = 0.024), antihypertensive agents use [odds ratio (OR) = 1.86; *p* = 0.041], 15-day Barthel Index score (OR = 0.930; *p* < 0.001) and 15-day mRS score (OR = 13.494; *p* < 0.001) were selected as predictors for the unfavorable outcome nomogram. The nomogram model showed good predictive performance in both the training (AUC = 0.950) and test cohorts (AUC = 0.942).

**Conclusion:**

The constructed nomogram model could be a practical tool for predicting unfavorable functional outcomes in ischemic stroke patients underwent early rehabilitation after acute phase.

## 1. Introduction

Annual data has shown that of the 16 million people who suffer from stroke worldwide, 87% had an ischemic stroke. More than 6 million deaths or disabilities are attributable to stroke, resulting in heavy social, family, and economic burdens. Reduction of disabilities and improvement of the independence of stroke survivors have been the primary goals of rehabilitation (Langhorne et al., 2011). Early identification of individuals at risk of an unfavorable outcome is important for decision-making in clinical practice and could help improve the disease outcome (Ali-Ahmed et al., 2019; Diener and Hankey, 2020). Predictive factors are not only used to predict prognosis but also stratify patients for individualized treatment (Gravanis and Tsirka, 2008; Diener and Hankey, 2020; Lopatkiewicz et al., 2020).

Previous research on risk prediction of outcome in ischemic stroke patients relied mostly on demographic variables, physical examination variables, disease-related variables, and laboratory and imaging variables (Fahey et al., 2018). The most commonly used risk factors in the prediction of functional outcomes included age, baseline National Institutes of Health Stroke Scale (NIHSS) score, stroke subtypes, and lesion size (Lucke-Wold et al., 2012; Yu et al., 2015), whereas mortality, recurrence rate, and complication rate were the target outcomes (Lin et al., 2019; You et al., 2019; Montellano et al., 2021; Noubiap et al., 2021). The majority of the available data used for prediction focused mostly on the acute phase of the disease, with few extended longitudinal follow-ups (Faura et al., 2021). Early rehabilitation efforts have been shown to have a positive effect on functional outcomes at 3 months, in acute stroke patients (Winstein et al., 2016; Ahmed et al., 2020). However, studies on diagnostics, rehabilitation, and prognostics of stroke recovery have not kept pace (Moradi et al., 2021; Preston et al., 2021). During this prolonged rehabilitation process, although there were intermittent functional assessments, prognosis judgment was mainly based on the experience of the physician.

Different nomograms have been developed to predict mortality, stroke-associated infections, malignant cerebral edema, post-stroke depression, hemorrhagic transformation, or unfavorable outcomes in stroke populations with different characteristics (Du et al., 2020; Lan et al., 2020, 2022; Szlachetka et al., 2022; Zhang C. et al., 2022; Zhang K. et al., 2022). Zhang C. et al. (2022) innovatively developed a dynamic nomogram to predict the 3-month unfavorable outcome of patients with acute ischemic stroke based on glycosylated hemoglobin, the Alberta Stroke Program Early Computed Tomography Score (ASPECTS), and NIHSS score at day 14, with C-index of 0.891 (95% CI, 0.854–0.928). However, this model still has some limitations, such as being limited by the sample size (*n* = 93), this model lacks external validation, and requires invasive blood draw and early Computed Tomography (CT) scan (Zhang C. et al., 2022).

We developed an effective and practical nomogram for predicting unfavorable functional outcomes following the acute phase using multidimensional data such as rehabilitation intervention-related features and longitudinally collected data from several Chinese institutions.

## 2. Materials and methods

### 2.1. Study design

This multicenter retrospective observational study was based on baseline data and longitudinally collected rehabilitation features extracted from various institutions in China. This retrospective study was approved by the Institutional Ethics Review Board, which waived the requirement for informed consent.

### 2.2. Patient selection

Ischemic stroke patients who underwent rehabilitation between August 2018 and November 2020 at these institutions were screened, and randomly allocated to either the training cohort or the test cohort in a ratio of 4:1. Patients diagnosed as acute ischemic stroke (within 24 h of onset) based on clinical and computed tomography findings, aged 18 years or older, and underwent early rehabilitation were included in this study. Patients with hemorrhagic stroke, transient ischemic attack, baseline NIHSS score <2, without modified Rankin Scale (mRS) score 90 days after stroke, and those with severe cognitive and mental dysfunction were excluded from this study.

### 2.3. Data collection

We collected baseline demographic data (such as age, gender, occupation, or education), laboratory and clinical examination data (such as hemoglobin A1c, triglycerides, or total cholesterol), pharmaceutical and invasive therapy-related data (such as the use of intravenous thrombolysis, endovascular therapy or antiplatelet therapy within 48 h) within 48 h after onset, and rehabilitation featured data (such as time from onset to first rehabilitation intervention, time from onset to first effective mobilization or total length of effective mobilization within first 14 days) on the 15th day after onset. In addition, longitudinal follow-up data such as the NIHSS score, Barthel Index (BI), and mRS score were collected 24 h and 15 days following ischemic stroke. The target outcome was functional status, which was assessed 90 days following onset using the mRS questionnaire. The favorable functional outcome was defined as an mRS score of 0–2, while the unfavorable outcome was an mRS score of 3–6 (Zheng et al., 2021).

### 2.4. Statistical analysis

**FIGURE 1:**
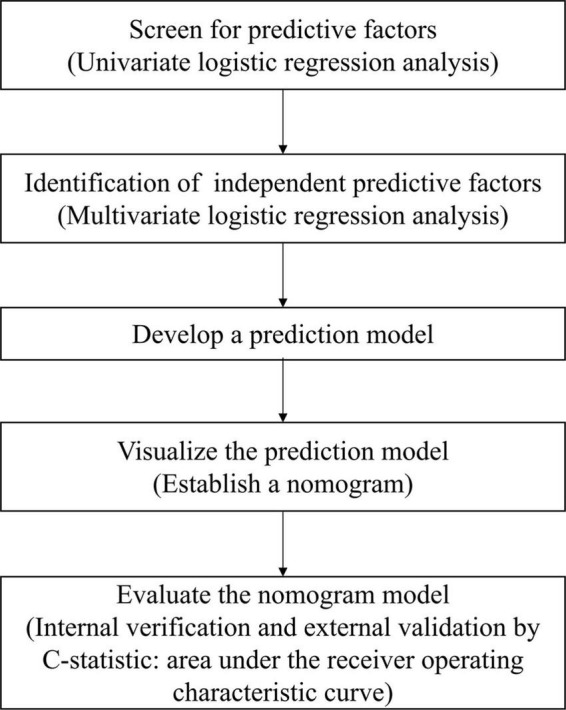
A flowchart displaying the process of building the nomogram.

All statistical analysis was performed using SPSS 19.0 software (SPSS Inc., Chicago, IL, USA). The missing data at baseline were managed with mean imputation, whereas missing data at follow-up were imputed using the last observation carried forward method (Kim et al., 2019). Baseline demographics were presented as a mean (standard deviation) or a number (percentage). Student’s *T*-test or Mann–Whitney *U* test was used to compare baseline data between the training cohort and the test cohort for continuous variables, while Chi-square or Fisher’s exact test was used for categorical variables. Variables with statistical significance (*p* < 0.05) in the univariate logistic regression were included in the multivariate logistic regression to identify predictors of unfavorable functional outcomes. The nomogram was developed using predictors in the training cohort identified by multivariate logistic regression. The nomogram was constructed using the regression modeling strategies package in R version 3.0.2 (R Project for Statistical Computing).^1^ After establishing the predictive nomogram, the C-statistic and the receiver operating characteristic (ROC) curve were used to validate the accuracy and discriminative ability of the nomogram both internally (training cohort) and externally (test cohort). The C-statistic was calculated as the area under the ROC curve (AUC) and was used to evaluate the predictive performance of the model. The optimal cut-off value for clinical use was determined by maximizing the Youden index (sensitivity + specificity-1). Statistical significance was defined by a two-tailed *p*-value of less than 0.05. Figure 1 shows a flowchart displaying the process of building the nomogram.

## 3. Results

### 3.1. Patient characteristics

A total of 856 ischemic stroke patients (684 in the training cohort and 172 in the test cohort) were included in our analysis. Our data revealed that the mean age of the participants was 63.73 years, and 585 of them were males (Table 1). A total of 518 participants experienced unfavorable outcomes 90 days following an ischemic stroke (Supplementary Table 1). There were no statistically significant differences between the training cohort and the test cohort in terms of demographic variables, Trial of ORG 10172 in Acute Stroke Treatment (TOAST) classification, or 90-day mRS proportion. The rate of missing data was less than 3.7% for all variables except education, which had a missing data rate of 9.1%. The overall missing data rate was approximately 0.7%.

**TABLE 1:** Clinical characteristics of ischemic stroke patients who underwent early rehabilitation.

	Overall (*n* = 856)	Training cohort (*n* = 684)	Test cohort (*n* = 172)	*p*-Value
Male, *n* (%)	585 (68.3)	467 (68.3)	118 (68.6)	0.934
Age, years, mean (SD)	63.73 (12.02)	63.67 (11.79)	63.97 (12.91)	0.778
Occupation, *n* (%)				0.342
Full-time or part-time paid work	167 (19.5)	127 (18.6)	40 (23.3)	
Layoffs	53 (6.2)	39 (5.7)	14 (8.1)	
Retired	369 (43.1)	299 (43.7)	70 (40.7)	
Self-employed	142 (16.6)	119 (17.4)	23 (13.4)	
Home duties	125 (14.6)	100 (14.6)	25 (14.5)	
Education, *n* (%)				0.698
Primary school or less	334 (39.0)	271 (14.5)	23 (13.4)	
Secondary school	221 (25.8)	169 (24.7)	52 (30.2)	
High school	133 (15.5)	108 (15.8)	25 (14.5)	
College/university	46 (5.4)	37 (5.4)	9 (5.2)	
Smoking index, cigarettes per day × year, mean (SD)	176.59 (346.67)	184.35 (363.39)	145.76 (268.83)	0.192
Alcohol intake, *n* (%)				0.903
No drinking	575 (67.2)	457 (66.8)	118 (68.6)	
Light drinking	178 (20.8)	144 (21.1)	34 (19.8)	
Heavy drinking	103 (12.0)	83 (12.1)	20 (11.6)	
Regular physical activities, *n* (%)				0.078
Yes	203 (23.7)	171 (25.0)	32 (18.6)	
No	653 (76.3)	513 (75.0)	140 (81.4)	
Medical history, *n* (%)				
Hypertension	564 (65.9)	442 (64.6)	122 (70.9)	0.119
Diabetes mellitus	229 (26.8)	118 (26.5)	48 (27.9)	0.702
Dyslipidemia	35 (4.1)	30 (4.4)	5 (2.9)	0.381
Atrial fibrillation	47 (5.5)	38 (5.6)	9 (5.2)	0.868
Coronary heart disease	68 (7.9)	53 (7.7)	15 (8.7)	0.673
Myocardial infarction	11 (1.3)	9 (1.3)	2 (1.2)	1.000
Congenital heart disease	3 (0.4)	2 (0.3)	1 (0.6)	1.000
Valvular heart disease	10 (1.2)	9 (1.3)	1 (0.6)	0.686
Body mass index, kg/m^2^, mean (SD)	24.46 (3.58)	24.46 (3.48)	24.47 (3.95)	0.974
Systolic blood pressure, mmHg, mean (SD)	151.75 (20.37)	151.80 (20.53)	151.56 (19.78)	0.893
Diastolic blood pressure, mmHg, mean (SD)	89.54 (14.23)	89.47 (14.09)	89.86 (14.80)	0.746
Heart rate, mean (SD)	77.54 (11.59)	77.62 (11.07)	77.25 (13.47)	0.740
Baseline NIHSS score, *n* (%)				0.803
NIHSS 2–7, mild	424 (49.5)	341 (49.9)	83 (48.3)	
NIHSS 8–16, moderate	390 (45.6)	311 (45.5)	79 (45.9)	
NIHSS >16, severe	42 (4.9)	32 (4.7)	10 (5.8)	
TOAST classification, *n* (%)				0.168
Large artery atherosclerosis	409 (47.8)	323 (47.2)	86 (50.0)	
Cardio embolism	48 (5.6)	38 (5.6)	10 (5.8)	
Small artery occlusion	332 (38.8)	276 (40.4)	56 (32.6)	
Stroke of other determined cause	42 (4.9)	29 (4.2)	13 (7.6)	
Stroke of undermined cause	25 (2.9)	18 (2.6)	7 (4.1)	
90-day mRS, *n* (%)				0.590
mRS 0–2	338 (39.5)	267 (39.0)	71 (41.3)	
mRS 3–6	518 (60.5)	417 (61.0)	101 (58.7)	

Frequencies and percentages are reported for categorical variables, while mean ± SD are reported for continuous variables. SD, standard deviation; NIHSS, National Institute of Health Stroke Scale; mRS, modified Rankin Scale.

### 3.2. Predictors of unfavorable functional outcomes

Overall, 45 variables were subjected to univariate analyses (Table 2). Among them, 17 variables with *p* < 0.05, including 5 demographic and anthropometric variables (age, occupation, atrial fibrillation history, coronary heart disease history, and myocardial infarction history), 2 laboratory and clinical examination variables (TOAST classification and hemoglobin A1c), 1 pharmaceutical and invasive therapy-related variable (antihypertensive agents), 3 rehabilitation variables (time from onset to first effective mobilization, effective mobilization in first rehabilitation intervention, a total length of effective mobilization within first 14 days in minutes), and 6 longitudinal follow-up data (NIHSS, BI, and mRS scores assessed at both baseline and 15-day) were selected as possible predictors.

**TABLE 2:** Correlation coefficients and odds ratios in the univariate logistic regression analysis of the training cohort.

	β	OR	*p*
**Demographic and anthropometric variables**			
Gender			
Female	/	/	
Male	−0.205	0.814	0.220
Age	0.023	1.023	0.001
Occupation			0.018
Full-time or part-time paid work	/	/	
Layoffs	0.234	1.264	0.551
Retired	0.555	1.742	0.015
Self-employed	0.466	1.593	0.087
Home duties	0.927	2.528	0.001
Education			0.080
Primary school or less	/	/	
Secondary school	0.069	1.071	0.774
High school	−0.208	0.812	0.424
College/university	0.090	1.094	0.75
Smoking index	0.000	1.000	0.391
Alcohol intake			0.497
No drinking	/	/	
Light drinking	0.104	1.109	0.593
Heavy drinking	−0.235	0.791	0.350
Regular physical activities			
Yes	/	/	
No	0.327	1.386	0.078
Hypertension history			
No	/	/	
Yes	−0.228	0.796	0.162
Diabetes mellitus history			
No	/	/	
Yes	0.292	1.338	0.097
Dyslipidemia history			
No	/	/	
Yes	−0.105	0.900	0.786
Atrial fibrillation history			
No	/	/	
Yes	0.697	2.009	0.038
Coronary heart disease history			
No	/	/	
Yes	0.774	2.168	0.007
Myocardial infarction history			
No	/	/	
Yes	2.553	12.849	0.016
Body mass index	0.021	1.022	0.338
Systolic blood pressure	0.005	1.005	0.197
Diastolic blood pressure	−0.009	0.991	0.107
Heart rate	−0.001	0.999	0.860
**Laboratory and clinical examination variables**			
TOAST classification			<0.001
Large artery atherosclerosis	/	/	
Cardio embolism	0.13	1.139	0.704
Small artery occlusion	−0.579	0.560	0.001
Stroke of other determined cause	−1.702	0.182	0.002
Stroke of undermined cause	−1.949	0.142	0.010
OCSP classification			0.088
Total anterior circulation infarction	/	/	
Partial anterior circulation infarction	−0.402	0.669	0.101
Posterior circulation infarction	−0.497	0.608	0.106
Lacunar infarction	−0.766	0.465	0.012
Hemoglobin A1c	0.106	1.112	0.016
Triglycerides	0.014	1.015	0.322
Total cholesterol	0.039	1.040	0.203
Low-density lipoprotein cholesterol	0.088	1.092	0.229
High-density lipoprotein cholesterol	−0.206	0.814	0.261
Homocysteine	0.001	1.001	0.862
Prothrombin time-international normalized ratio	−0.145	0.865	0.125
Activated partial thromboplastin time	0.005	1.005	0.669
**Pharmaceutical and invasive therapy related variables**			
Intravenous thrombolysis			
Yes	/	/	
No	0.125	1.134	0.487
Endovascular therapy			
Yes	/	/	
No	0.074	1.077	0.820
Antiplatelet therapy within 48 h			
Yes	/	/	
No	0.333	1.395	0.199
Anticoagulant therapy within 48 h			
Yes	/	/	
No	0.225	1.252	0.257
Antihypertensive agents			
Yes	/	/	
No	0.443	1.558	0.006
Lipid regulators			
Yes	/	/	
No	−0.383	0.682	0.115
Hypoglycemic agents			
Yes	/	/	
No	−0.114	0.892	0.524
**Rehabilitation featured variables**			
Time from onset to first rehabilitation intervention	0.000	1.000	0.957
Time from onset to first effective mobilization	0.006	1.006	<0.001
Effective mobilization in first rehabilitation intervention			
Yes	/	/	
No	0.665	1.945	<0.001
Length of effective mobilization in first rehabilitation intervention (minutes)	−0.014	0.986	0.062
Total length of effective mobilization within first 14 days (minutes)	−0.001	0.999	0.001
**Longitudinal follow-up variables**			
NIHSS score at baseline	0.149	1.161	<0.001
15-day NIHSS	0.274	1.315	<0.001
Barthel Index score at baseline	−0.073	0.930	<0.001
15-day Barthel Index	−0.089	0.915	<0.001
mRS score at baseline	2.035	7.655	<0.001
15-day mRS	3.278	26.520	<0.001

β, correlation coefficient; OR, odds ratio; TOAST, Trial of ORG 10172 in Acute Stroke Treatment; OCSP, Oxfordshire Community Stroke Project; NIHSS, National Institute of Health Stroke Scale; mRS, modified Rankin Scale.

Multivariate logistic regression analysis revealed that TOAST classification (*p* = 0.024), antihypertensive agents use [odds ratio (OR) = 1.86; 95% CI: 1.026, 3.373; *p* = 0.041], 15-day BI score (OR = 0.930; 95% CI: 0.902, 0.960; *p* < 0.001), and 15-day mRS score (OR = 13.494; 95% CI: 6.871, 26.501; *p* < 0.001) were significant predictors. Using large artery atherosclerosis (LAA) subtype as reference, stroke of other determined cause (OR = 0.105; 95% CI: 0.015, 0.720; *p* = 0.022) and stroke of undermined cause (OR = 0.038; 95% CI: 0.003, 0.483; *p* = 0.012) subtypes exhibited a lower risk (Table 3).

**TABLE 3:** Multivariate logistic regression for unfavorable functional outcomes in the training cohort.

	β	*p*	OR (95% CI)
Age	0.014	0.400	1.014 (0.982, 1.047)
Occupation		0.237	
Full-time or part-time paid work	/	/	
Layoffs	0.094	0.902	1.098 (0.245, 4.932)
Retired	−0.004	0.994	0.996 (0.361, 2.749)
Self-employed	0.091	0.859	1.095 (0.402, 2.984)
Home duties	1.069	0.072	2.913 (0.910, 9.324)
**Atrial fibrillation history**			
No	/	/	
Yes	0.681	0.483	1.977 (0.294, 13.291)
**Coronary heart disease history**			
No	/	/	
Yes	0.863	0.156	2.371 (0.719, 7.811)
**Myocardial infarction history**			
No	/	/	
Yes	6.000	0.100	403.35 (0.317, 512636.336)
TOAST classification		0.024	
Large artery atherosclerosis	/	/	
Cardio embolism	0.158	0.852	1.171 (0.223, 6.148)
Small artery occlusion	−0.090	0.781	0.914 (0.483, 1.726)
Stroke of other determined cause	−2.258	0.022	0.105 (0.015, 0.720)
Stroke of undermined cause	−3.273	0.012	0.038 (0.003, 0.483)
Hemoglobin A1c	0.140	0.082	1.150 (0.982, 1.347)
**Antihypertensive agents**			
Yes	/	/	
No	0.621	0.041	1.86 (1.026, 3.373)
Time from onset to first effective mobilization	0.002	0.351	1.002 (0.998, 1.006)
**Effective mobilization in first rehabilitation intervention**			
Yes	/	/	
No	−0.636	0.098	0.529 (0.249, 1.124)
Total length of effective mobilization within first 14 days (minutes)	0.000	0.835	1.000 (0.998, 1.001)
NIHSS score at baseline	0.027	0.675	1.028 (0.905, 1.167)
15-day NIHSS	−0.035	0.668	0.965 (0.822, 1.134)
Barthel Index score at baseline	0.011	0.419	1.011 (0.984, 1.039)
15-day Barthel Index	−0.072	<0.001	0.930 (0.902, 0.960)
Baseline mRS score	0.598	0.051	1.819 (0.996, 3.320)
15-day mRS	2.602	<0.001	13.494 (6.871, 26.501)
Intercept	−10.670	<0.001	
Nagelkerke *R*^2^	0.773		

β, correlation coefficient; OR, odds ratio; CI, confidence interval; TOAST, Trial of ORG 10172 in Acute Stroke Treatment; NIHSS, National Institute of Health Stroke Scale; mRS, modified Rankin Scale.

According to the ROC curve analyses, the TOAST classification and antihypertensive agents use did not perform significantly well when used for individual prediction. The AUC value of TOAST classification in the test cohort was 0.589 (95% CI: 0.503, 0.674), while the AUC value of antihypertensive agents use was 0.536 (95% CI: 0.447, 0.625). In contrast, the 15-day BI score had an AUC value of 0.868 (95% CI: 0.815, 0.921), and the 15-day mRS score had an even better AUC value of 0.903 (95% CI: 0.858, 0.948) in the test cohort, indicating their good predictive performance (Supplementary Figure 1).

### 3.3. Development and validation of the nomogram

**FIGURE 2:**
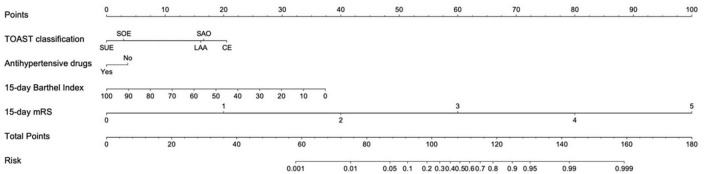
Nomogram for predicting unfavorable functional outcome 90 days after ischemic stroke. LAA, large artery atherosclerosis; CE, cardio embolism; SAO, small artery occlusion; SOE, stroke of other determined cause; SUE, stroke of undermined cause. Antihypertensive agents use refers to whether antihypertensive agents were used within 48 h after the onset of ischemic stroke. The cut-off value for an unfavorable functional outcome risk was 0.45.

Based on the predictors, a nomogram was developed to predict unfavorable functional outcomes (Figure 2). In the nomogram, each patient received a total score based on baseline characteristics (TOAST classification and antihypertensive agents) and 15-day follow-up characteristics (15-day BI score and 15-day mRS score) to predict the occurrence of unfavorable functional outcomes with risk percentages. According to the maximized Youden index, the optimal cut-off value for an unfavorable functional outcome risk was 0.45.

**FIGURE 3:**
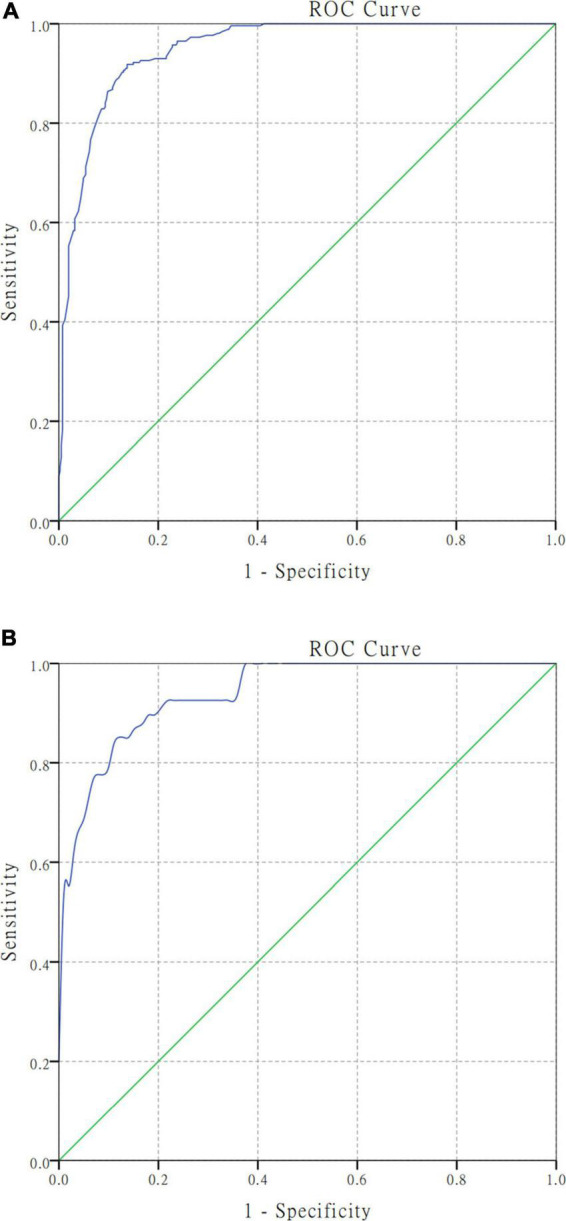
Receiver operating characteristic curve analyses for the nomogram model in the training and test cohort. Harrell’s C−statistic was calculated as the AUC (area under the curve) value. **(A)** The AUC was 0.950 (95% CI, 0.935–0.965) in the training cohort for predicting unfavorable functional outcomes. **(B)** The AUC was 0.942 (95% CI, 0.910–0.974) in the test cohort for predicting unfavorable functional outcomes.

The AUC of the model in the training cohort was 0.950 (95% CI, 0.935–0.965; Figure 3A), while that of the test cohort was 0.942 (95% CI, 0.910–0.974; Figure 3B).

## 4. Discussion

Early rehabilitation is considered an important treatment after an ischemic stroke and has been widely accepted in stroke units. Previous studies have demonstrated that early rehabilitation has positive effects on poststroke disability and health-related quality of life after ischemic stroke (Askew et al., 2020; Huang et al., 2020), however, there are no practical tool predicting the long-term functional outcomes during the rehabilitation phase. Previous predictive tools have focused on predicting outcomes before intervention, which has aided in the development of early treatment decision-making, such as thrombolysis, mechanical thrombectomy or rehabilitation treatment plans (Ang et al., 2003; Cho et al., 2008; Lan et al., 2020; Zhang C. et al., 2022). However, it is important to note that the recovery process after ischemic stroke is a long-term and continuous process. As such, the rehabilitation treatment plan may require continuous adjustments, and evaluation and new predictions serve as the basis for adjusting the plan. Our nomogram predicts long-term functional outcomes for patients 15 days after onset, providing more accurate predictions for physicians and patients during this period and helping to adjust subsequent treatment plans. Unlike previous tools, our tool is designed for patients who have undergone treatment for a period of time, rather than those with recent onset of ischemic stroke. A significant advantage of our tool is that the variables required for prediction are easily obtainable. Only BI and mRS scale assessments are required 15 days after onset, combined with retrospective collection of antihypertensive drug use and TOAST classification results. Our tool does not require invasive examinations or strong dependence on magnetic resonance imaging results, making it easier to promote and use compared to previous tools.

Previous research suggests that stroke severity (NIHSS score), age, gender, and other factors all influence stroke prognosis (Clua-Espuny et al., 2019). However, few studies have focused on features of rehabilitation therapy and prediction aiding in the design and adjustment of rehabilitation programs. As patients’ health conditions improve, a growing number of them receive early rehabilitation and continuous follow-up, which has made it possible to use rehabilitation characteristics to predict the risk of unfavorable functional outcomes in this population.

Similar to previous studies (Zhang et al., 2019), this study discovered that LAA (47.8%) and small artery occlusion (SAO) (38.8%) were the predominant subtypes in ischemic stroke. Studies have shown that the severity and prognosis of stroke patients are correlated with the TOAST classification (Leira et al., 2008). Cardio embolism (CE) subtype had the highest risk, whereas SAO and LAA subtypes had a higher risk than a stroke caused by other specified causes and stroke of undermined cause subtypes. This may be because the severity of the CE subtype is relatively severe, and its prognosis is worse, which is likely due to underlying cardiac pathologies such as arrhythmias and heart failure. A study by Bjerkreim et al. (2019) revealed that the 5-year risk of mortality and readmission for all causes was significantly higher in patients with CE compared with other subtypes. Chen et al. (2022) performed an outcome prediction of 10,967 patients with transient ischemic attack and minor stroke, and found that the TOAST classification was the influencing factor, the risk of LAA subtype was the highest, and the risk of SAO subtype was the lowest. A few studies have been conducted on people undergoing rehabilitation, disparate outcomes are likely attributable to distinct populations. However, in general, early assessment of TOAST classification in patients with ischemic stroke is of great significance for secondary prevention and prediction of poor prognosis.

In this retrospective study, the use of antihypertensive agents within 48 h after ischemic stroke was associated with a higher risk of unfavorable outcomes (OR = 1.86). However, neither systolic nor diastolic blood pressure (BP) levels were significant predictors. The management of hypertension in the acute stage of an ischemic stroke remains controversial, as observational studies are unable to determine whether elevated BP level is a poor prognostic factor following acute ischemic stroke (Ntaios et al., 2010, 2011). Following an acute ischemic stroke, prospective trials are necessary to validate the timing of BP lowering and choice of agent. As reported, acute and aggressive BP lowering within 24 h of stroke onset could jeopardize the outcome (Georgianou et al., 2018) and the benefit of starting antihypertensive therapy within 48 or 72 h after onset remains unclear (He et al., 2014). For patients not receiving intravenous recombinant tissue-type plasminogen activator (rt-PA), endovascular treatment and with no compelling medical condition to dictate acute BP lowering therapy, BPs up to 220/120 mmHg may be observed without BP lowering therapy in the first several days according to the latest American Heart Association/American Stroke Association [AHA/ASA] (2022) guidelines. At the same time, the AHA/ASA guidelines recommended that BP should be controlled to a level <185/110 mmHg before intervention and maintained at levels <180/105 mmHg in patients who require administration of rt-PA, intra-arterial fibrinolysis or mechanical thrombectomy (Powers et al., 2018). The Enhanced Control of Hypertension and Thrombolysis Stroke Study, on the other hand, found that achieving early and persistently low levels of SBP <140 mmHg within 24 h, even as low as 110–120 mmHg, was associated with better outcomes in thrombolytic-eligible acute ischemic stroke patients (Wang et al., 2022). Our findings suggest a potential benefit of antihypertensive agent use, but because this was an observational study, we cannot conclude that there is a direct causal relationship between antihypertensive agent use and outcomes. The deep relationship requires additional investigation. This study included patients who received thrombolysis, thrombectomy, and conservative treatment, limiting further interpretation of the results.

The NIHSS score is the world’s most widely used acute ischemic stroke scale (Saber and Saver, 2020). A previous study reported that discharged patients with high NIHSS scores were generally more severely ill and had larger brain infarct volumes, which were associated with 90-day outcomes in ischemic stroke patients (Wouters et al., 2018). In this study, the NIHSS score was also a potential influencing factor, although it was not included in the final model. The mRS score is widely used to assess disability; the higher the score, the higher the risk of unfavorable outcomes. The BI is widely used to assess daily activities of living, and the higher the score, the lower the risk of poor outcomes. The NIHSS, mRS, and BI are all associated with more severe disease, a larger infarct volume in brain tissue, and a 90-day prognosis in ischemic stroke patients (Govan et al., 2009). Unlike the results of Zhang C. et al. (2022), the NIHSS score was not included in our final model, which could be attributed to the increased contribution of mRS and BI scores. Furthermore, the NIHSS is an 11-item scale that assesses consciousness, vision, language, sensory, and motor function. The lack of strong correlation between NIHSS and outcomes may be due to the existence of multiple subscales. This could be the reason why NIHSS does not perform as well as BI and mRS in predicting 90-day mRS score (Schmid et al., 2011). At the same time, the mRS and BI scores at 15 days were more valuable in predicting outcome than the mRS and BI scores at baseline. Similarly, Chen et al. (2022) found that discharge mRS and discharge NIHSS score were associated with a poor 90-day prognosis, with discharge mRS having a greater predictive contribution than admission mRS. These findings indicated the importance of follow-up data in outcome prediction. Therefore, we emphasize the importance of reassessment after the acute phase, such as 15 days after onset.

There are some advantages and limits to this study. The first strength is that this study provides a basis for predicting the prognosis of patients in the rehabilitation stage. Second, although our study only used four predictors for modeling, the model and nomogram built by logistic regression achieved great prediction performance and were more convenient for external validation and nomogram use due to the small number and easy to obtain of features. The following are the limitations. First, this is a retrospective study and it is subject to the selection and follow-up bias. Second, we included the variables selected by univariate logistic regression in the multivariate logistic regression, which may lead to underestimation of interactions between variables. Third, the data included in this study is from patients who received early rehabilitation, limiting the generalizability of our findings to countries with lower rates of patients receiving early rehabilitation. As stated previously, however, an increasing number of stroke patients can receive early rehabilitation following the acute phase, and this population is growing. Fourth, our nomogram uses 15-day follow-up data, therefore patients lacking follow-up data will be limited to use this nomogram. Finally, the model can also incorporate machine learning methods to increase the accuracy of future predictions.

Future studies will use rehabilitation-related features to make more precise predictions of long-term functions such as swallowing function, walking ability, and aphasia. The unresolved question is how to identify individuals who can transition from higher mRS levels to lower mRS levels.

## 5. Conclusion

In conclusion, the nomogram can accurately predict the risk of unfavorable functional outcomes in the early rehabilitation population 90 days after ischemic stroke, with longitudinally collected data and rehabilitation-specific characteristics such as the 15-day mRS score and the 15-day BI score, serving as the predominant predictors. Patients at high risk (>0.45) should receive additional care to prevent unfavorable outcomes.

## Data availability statement

The raw data supporting the conclusions of this article will be made available by the authors, without undue reservation.

## Ethics statement

The studies involving human participants were reviewed and approved by the Ethics Committee of the First Affiliated Hospital of Nanjing Medical University. Written informed consent for participation was not required for this study in accordance with the national legislation and the institutional requirements.

## Author contributions

CY, YZhe, XZ, CG, and XLu contributed to the conception and design of the study. SW, YZhu, YJ, XLi, GF, HP, MT, JL, and KQ collected the data. CY and LX performed the statistical analysis. CY drafted the manuscript. YZhe and XLu revised the manuscript. All authors contributed to the article and approved the submitted version.

## Footnotes

1
www.r-project.org
