# Supplementary material for: Development and validation of a nomogram model for predicting unfavorable functional outcomes in ischemic stroke patients after acute phase
Source: Front Aging Neurosci. 2023 Jul 14;15:1161016. doi: 10.3389/fnagi.2023.1161016 (PMC10375043; doi:10.3389/fnagi.2023.1161016)
Supplement: Supplementary file 1 [file Data_Sheet_1.docx]

Development and validation of a nomogram model for predicting unfavorable functional outcomes in ischemic stroke patients after acute phase

# Supplementary Figures and Tables

## Supplementary Figures

**Figure S1: Receiver operating characteristic curve analyses for the independent predictors in the training and test cohort**


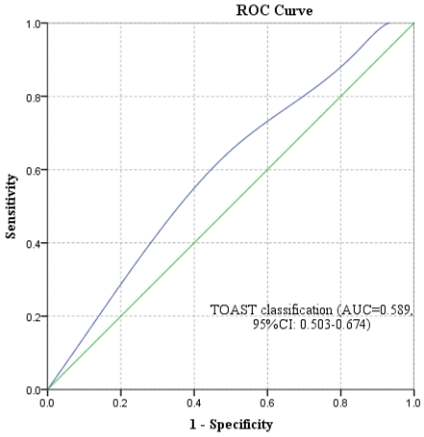

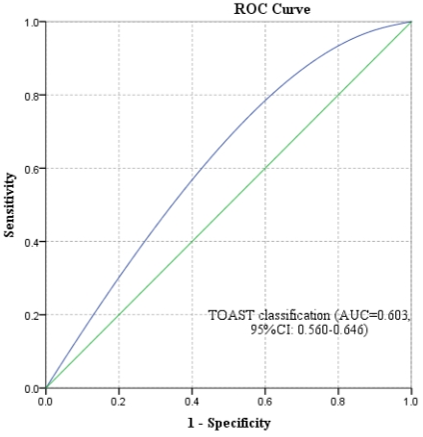


(B)

(A)


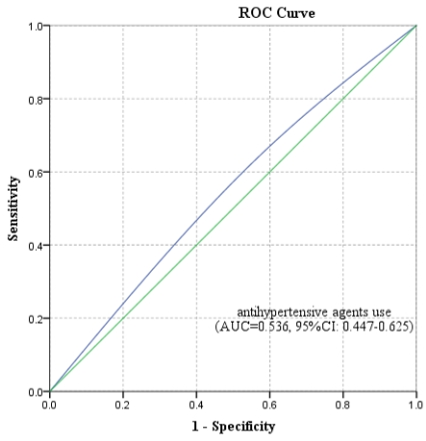

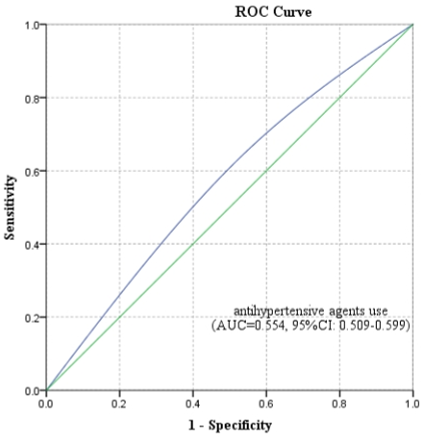


(D)

(C)


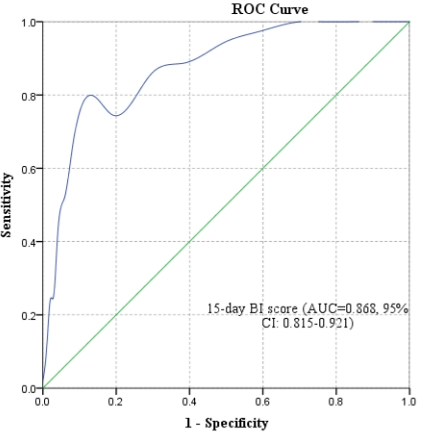

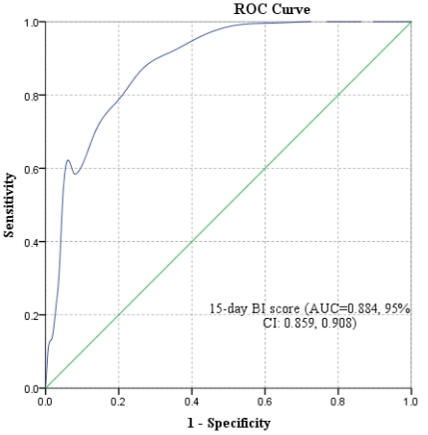


(F)

(E)


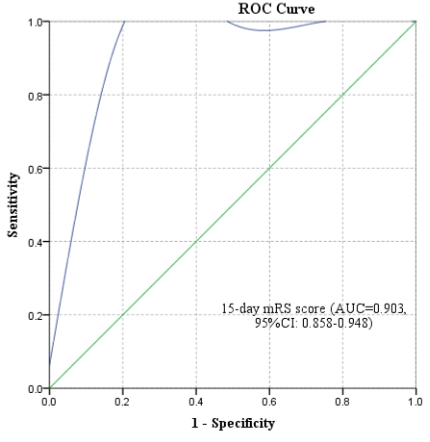

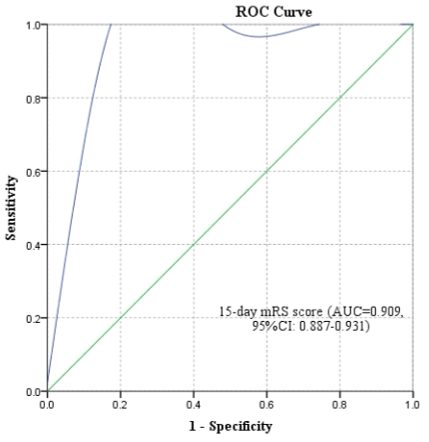


(H)

(G)

**Figure S1: Receiver operating characteristic curve analyses for the independent predictors in the training and test cohort**
ROC: Receiver operating characteristic. (**A**) The ROC curve in the training cohort of model based on TOAST classification. (**B**) The ROC curve in the test cohort of model based on TOAST classification. (**C**) The ROC curve in the training cohort of model based on antihypertensive agents use. (**D**) The ROC curve in the test cohort of model based on antihypertensive agents use. (**E**) The ROC curve in the training cohort of model based on 15-day BI score. (**F**) The ROC curve in the test cohort of model based on 15-day BI score. (**G**) The ROC curve in the training cohort of model based on 15-day mRS score. (**H**) The ROC curve in the test cohort of model based on 15-day mRS score.

## Supplementary Tables

**Table S1: Clinical characteristics of ischemic stroke patients with favorable outcomes and unfavorable outcomes**

|  | Overall (n=856) | Favorable outcome (n=518) | Unfavorable outcome (n=338) | *p Value* |
| --- | --- | --- | --- | --- |
| **Demographic and Anthropometric Variables** |  |  |  |  |
| Gender |  |  |  | 0.230 |
| Female | 271 (31.7) | 156 (30.1) | 115 (34.0) |  |
| Male | 585 (68.3) | 362 (69.9) | 223 (66.0) |  |
| Age | 63.73 (12.02) | 62.69 (11.68 ) | 65.31 (12.37 ) | 0.002 |
| Occupation |  |  |  | 0.038 |
| Full-time or part-time paid work | 167 (19.5) | 116 (22.4) | 51 (15.1) |  |
| Layoffs | 53 (6.2) | 34 (6.6) | 19 (5.6) |  |
| Retired | 369 (43.1) | 217 (41.9) | 152 (45.0) |  |
| Self-employed | 142 (16.6) | 86 (16.6) | 56 (16.6) |  |
| Home duties | 125 (14.6) | 65 (12.5) | 60 (17.8) |  |
| Education |  |  |  | 0.061 |
| Primary school or less | 334 (39) | 194 (37.5) | 140 (41.4) |  |
| Secondary school | 221 (25.8) | 137 (26.4) | 84 (24.9) |  |
| High school | 133 (15.5) | 79 (15.3) | 54 (16) |  |
| College/University | 46 (5.4) | 37 (7.1) | 9 (2.7) |  |
| Postgraduate |  |  |  |  |
| Smoking Index | 176.59 (346.67) | 185.34 (352.96 ) | 163.19 (336.87 ) | 0.361 |
| Alcohol intake |  |  |  | 0.126 |
| No drinking | 575 (67.2) | 346 (66.8) | 229 (67.8) |  |
| Light drinking | 178 (20.8) | 101 (19.5) | 77 (22.8) |  |
| Heavy drinking | 103 (12) | 71 (13.7) | 32 (9.5) |  |
| Regular physical activities |  |  |  | 0.031 |
| Yes | 203 (23.7) | 136 (26.3) | 67 (19.8) |  |
| No | 653 (76.3) | 382 (73.7) | 271 (80.2) |  |
| Hypertension history |  |  |  | 0.199 |
| No | 292 (34.1) | 168 (32.4) | 124 (36.7) |  |
| Yes | 564 (65.9) | 350 (67.6) | 214 (63.3) |  |
| Diabetes mellitus history |  |  |  | 0.175 |
| No | 627 (73.2) | 388 (74.9) | 239 (70.7) |  |
| Yes | 229 (26.8) | 130 (25.1) | 99 (29.3) |  |
| Dyslipidemia history |  |  |  | 0.520 |
| No | 821 (95.9) | 495 (95.6) | 326 (96.4) |  |
| Yes | 35 (4.1) | 23 (4.4) | 12 (3.6) |  |
| Atrial fibrillation history |  |  |  | 0.048 |
| No | 809 (94.5) | 496 (95.8) | 313 (92.6) |  |
| Yes | 47 (5.5) | 22 (4.2) | 25 (7.4) |  |
| Coronary heart disease history |  |  |  | 0.009 |
| No | 788 (92.1) | 487 (94) | 301 (89.1) |  |
| Yes | 68 (7.9) | 31 (6) | 37 (10.9) |  |
| Myocardial infarction history |  |  |  | 0.023 |
| No | 845 (98.7) | 515 (99.4) | 330 (97.6) |  |
| Yes | 11 (1.3) | 3 (0.6) | 8 (2.4) |  |
| Body mass index | 24.46 (3.58) | 24.32 (3.27 ) | 24.67 (4.00 ) | 0.164 |
| Systolic blood pressure | 151.75 (20.37) | 150.94 (19.64 ) | 152.99 (21.40 ) | 0.148 |
| Diastolic blood pressure | 89.54 (14.23) | 90.17 (14.73 ) | 88.59 (13.39 ) | 0.113 |
| Heart rate | 77.54 (11.59) | 77.69 (11.23 ) | 77.31 (12.12 ) | 0.640 |
| **Laboratory and Clinical Examination Variables** |  |  |  |  |
| TOAST classification |  |  |  | <0.001 |
| Large artery atherosclerosis | 409 (47.8) | 217 (41.9) | 192 (56.8) |  |
| Cardio embolism | 48 (5.6) | 24 (4.6) | 24 (7.1) |  |
| Small artery occlusion | 332 (38.8) | 221 (42.7) | 111 (32.8) |  |
| Stroke of other determined cause | 42 (4.9) | 33 (6.4) | 9 (2.7) |  |
| Stroke of undermined cause | 25 (2.9) | 23 (4.4) | 2 (0.6) |  |
| OCSP classification |  |  |  | 0.015 |
| Total anterior circulation infarction | 103 (12) | 50 (9.7) | 53 (15.7) |  |
| Partial anterior circulation infarction | 490 (57.2) | 294 (56.8) | 196 (58) |  |
| Posterior circulation infarction | 123 (14.4) | 78 (15.1) | 45 (13.3) |  |
| Lacunar infarction | 140 (16.4) | 96 (18.5) | 44 (13) |  |
| Hemoglobin A1c | 6.79 (1.87) | 6.69 (1.81 ) | 6.95 (1.93 ) | 0.046 |
| Triglycerides | 2.03 (6.31) | 1.86 (1.55 ) | 2.29 (9.85 ) | 0.331 |
| Total cholesterol | 4.56 (2.45) | 4.46 (1.40 ) | 4.71 (3.49 ) | 0.213 |
| Low-density lipoprotein cholesterol | 2.82 (3.67) | 2.61 (0.85 ) | 3.14 (5.74 ) | 0.094 |
| High-density lipoprotein cholesterol | 1.29 (2.95) | 1.39 (3.78 ) | 1.14 (0.42 ) | 0.222 |
| Homocysteine | 16.33 (12.12) | 16.17 (12.58 ) | 16.59 (11.38 ) | 0.622 |
| Prothrombin time-international normalized ratio | 1.22 (1.05) | 1.28 (1.21 ) | 1.13 (0.71 ) | 0.022 |
| Activated partial thromboplastin time | 28.37 (6.73) | 28.34 (6.90 ) | 28.41 (6.48 ) | 0.875 |
| **Pharmaceutical and Invasive Therapy related Variables** |  |  |  |  |
| Intravenous thrombolysis |  |  |  | 0.219 |
| Yes | 204 (23.8) | 123 (23.7) | 81 (24) |  |
| No | 645 (75.4) | 393 (75.9) | 252 (74.6) |  |
| Endovascular therapy |  |  |  | 0.393 |
| Yes | 51 (6) | 30 (5.8) | 21 (6.2) |  |
| No | 795 (92.9) | 484 (93.4) | 311 (92) |  |
| Antiplatelet therapy within 48h |  |  |  | 0.136 |
| Yes | 766 (89.5) | 472 (91.1) | 294 (87) |  |
| No | 85 (9.9) | 44 (8.5) | 41 (12.1) |  |
| Anticoagulant therapy within 48h |  |  |  | 0.208 |
| Yes | 165 (19.3) | 109 (21) | 56 (16.6) |  |
| No | 670 (78.3) | 395 (76.3) | 275 (81.4) |  |
| Antihypertensive agents |  |  |  | 0.004 |
| Yes | 359 (41.9) | 239 (46.1) | 120 (35.5) |  |
| No | 472 (55.1) | 268 (51.7) | 204 (60.4) |  |
| Lipid regulators |  |  |  | 0.047 |
| Yes | 728 (85) | 433 (83.6) | 295 (87.3) |  |
| No | 112 (13.1) | 78 (15.1) | 34 (10.1) |  |
| Hypoglycemic agents |  |  |  | 0.284 |
| Yes | 236 (27.6) | 141 (27.2) | 95 (28.1) |  |
| No | 594 (69.4) | 365 (70.5) | 229 (67.8) |  |
| **Rehabilitation Featured Variables** |  |  |  |  |
| Time from onset to first rehabilitation intervention | 51.33 (50.20) | 50.70 (55.41 ) | 52.30 (41.02 ) | 0.650 |
| Time from onset to first effective mobilization | 108.43 (311.66) | 80.35 (69.88 ) | 151.47 (485.67 ) | 0.008 |
| Effective mobilization in first rehabilitation intervention |  |  |  | <0.001 |
| Yes | 387 (45.2) | 272 (52.5) | 115 (34.0) |  |
| No | 438 (51.2) | 227 (43.8) | 211 (62.4) |  |
| Length of effective mobilization in first rehabilitation intervention (minutes) | 13.45 (11.20) | 14.20 (10.34 ) | 12.29 (12.32 ) | 0.015 |
| Total length of effective mobilization within first 14 days (minutes) | 248.81 (220.18) | 275.33 (210.73 ) | 208.17 (228.32 ) | <0.001 |
| **Longitudinal Follow-up Variables** |  |  |  |  |
| NIHSS score at baseline | 8.32 (4.61) | 7.11 (4.16 ) | 10.17 (4.65 ) | <0.001 |
| 15-day NIHSS | 6.13 (4.09) | 4.64 (3.47 ) | 8.41 (3.90 ) | <0.001 |
| Barthel Index score at baseline | 41.21 (22.66) | 51.04 (21.38 ) | 26.14 (15.02 ) | <0.001 |
| 15-day Barthel Index | 57.64 (23.91) | 69.86 (19.84 ) | 38.92 (16.30 ) | <0.001 |
| mRS score at baseline | 3.49 (1.05) | 3.05 (1.09 ) | 4.16 (0.49 ) | <0.001 |
| 15-day mRS | 3.00 (1.18) | 2.35 (1.08 ) | 3.98 (0.39 ) | <0.001 |

Frequencies and percentages are reported for categorical variables, while mean ± SD are reported for continuous variables. SD: standard deviation; NIHSS: National Institute of Health Stroke Scale; mRS: modified Rankin Scale. Student’s T test or Mann-Whitney U test was used to compare baseline data between the training cohort and the validation cohort for continuous variables, while Chi-square or Fisher's exact test was used for categorical variables.
